# Purification, Characterization and Biological Activity of Polysaccharides from *Dendrobium officinale*

**DOI:** 10.3390/molecules21060701

**Published:** 2016-05-30

**Authors:** Kaiwei Huang, Yunrong Li, Shengchang Tao, Gang Wei, Yuechun Huang, Dongfeng Chen, Chengfeng Wu

**Affiliations:** 1 College of Chinese Materia Medica, Guangzhou University of Chinese Medicine, Guangzhou 510006, China; huangkaiweifeng@163.com (K.H.); YYLi0711025@163.com (Y.L.); Taoshengchang2013@163.com (S.T.); W18719138511@hotmail.com (C.W.); 2 The First Affiliated Hospital of Guangzhou University of Traditional Chinese Medicine, Guangzhou 510080, China; 3 Department of Anatomy, Guangzhou University of Chinese Medicine, Guangzhou 510006, China; Cdf27212@21cn.com

**Keywords:** *Dendrobium officinale*, polysaccharide, structural characterization, immunostimulatory activity, antioxidant activity

## Abstract

Polysaccharide (DOPA) from the stem of *D. officinale*, as well as two fractions (DOPA-1 and DOPA-2) of it, were isolated and purified by DEAE cellulose-52 and Sephacryl S-300 chromatography, and their structural characteristics and bioactivities were investigated. The average molecular weights of DOPA-1 and DOPA-2 were 394 kDa and 362 kDa, respectively. They were mainly composed of d-mannose, d-glucose, and had a backbone consisting of 1,4-linked β-d-Man*p* and 1,4-linked β-d-Glc*p* with *O-*acetyl groups. Bioactivity studies indicated that both DOPA and its purified fractions (DOPA-1 and DOPA-2) could activate splenocytes and macrophages. The *D. officinale* polysaccharides had stimulatory effects on splenocytes, T-lymphocytes and B-lymphocytes, promoting the cell viability and NO production of RAW 264.7 macrophages. Furthermore, DOPA, DOPA-1 and DOPA-2 were found to protect RAW 264.7 macrophages against hydrogen peroxide (H_2_O_2_)-induced oxidative injury by promoting cell viability, suppressing apoptosis and ameliorating oxidative lesions. These results suggested that *D. officinale* polysaccharides possessed antioxidant activity and mild immunostimulatory activity.

## 1. Introduction

*Dendrobium officinale* (*D. officinale*), well known as *Tiepi Shihu*, is a precious traditional Chinese medicine in China. It is recorded in the Chinese Pharmacopoeia [1], and mainly distributed in the South of China [2]. *D. officinale* is traditionally recognized by traditional Chinese medical practitioners as the best *Dendrobium* herb for tonic purposes, and it has been used to alleviate diabetes, obesity, rheumatoid arthritis, and many other disease [3]. Because of its broad spectrum of medical properties, it is widely used as an ingredient in pharmaceuticals, nutraceuticals and food products. Currently, over-exploitation and habitat damage have caused serious scarcity of wild resources. Therefore, it was listed on the China Plant Red Data Book in 1987 [4]. The increasing demand and the short supply of the plant led to a dramatic rise in its price. Because of its potentially significant functions and high price, more and more researchers have paid attention to the study of *D. officinale*.

It has been reported that the main active ingredients of *D. officinale* include phenols, alkaloids, bibenzyls, terpenes, flavonoids, amino acids and polysaccharides [5,6,7,8,9,10]. Pharmacological studies have demonstrated that polysaccharides extracted from *D. officinale* possessed various biological activities, such as antioxidant, immunological, anti-tumour, hypoglycaemic activities and improvement of colonic health [11,12,13,14,15,16,17]. During the past 20 years, it had been found that most of the purified polysaccharides from *D. officinale* were isolated by DEAE cellulose-52 chromatography with water elution. The chemical characterization and the bioactivities of these purified polysaccharides had been studied [10,18,19,20,21,22]. These studies indicated that they had different structural characteristics and possessed antioxidant and immunostimulatory functions [19,21,22]. However, few studies have focused on the purified polysaccharides of *D. officinale* isolated by DEAE cellulose-52 chromatography with NaCl elution. Only one study reported that DOP-2 from *D. officinale* was isolated by DEAE cellulose-52 chromatography with NaCl elution and further purified by Sephacryl S-400 chromatography. The study mainly focused on the research of bioactivities of DOP-2. The evaluation of its bioactivities showed that DOP-2 had significant immunomodulatory activity *in vitro* [22]. However, the structural features and structure–activity relationship remain unknown.

In the present study, DOPA was isolated from *D. officinale* by DEAE cellulose-52 chromatography with NaCl elution. DOPA was further purified by Sephacryl S-300 chromatography, and two new polysaccharides (DOPA-1 and DOPA-2) were obtained. Therefore, the aims of this study were to preliminarily characterize the structure of the polysaccharide fractions isolated from the stem of *D. officinale* and to evaluate the bioactivity of these fractions *in vitro*.

## 2. Results

### 2.1. Extraction, Purification and Preliminary Characterization of DOPA Fractions

#### 2.1.1. Extraction and Purification of DOPA Fractions

**Figure 1:**
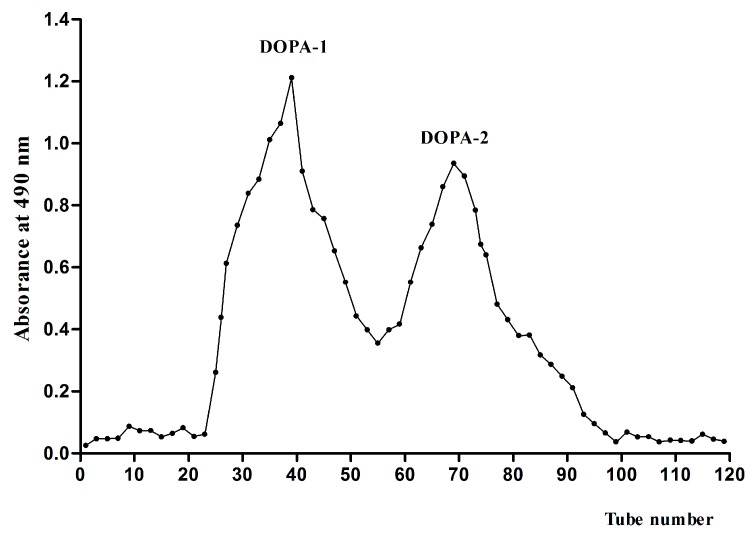
Elution profile of DOPA on a Sephacryl S-300 gel-permeation chromatography column.

The crude polysaccharides were passed through a DEAE-52 cellulose column and eluted with water, 0.1, 0.3, and 1.0 mol/L NaCl solutions, and then four fractions were obtained. Two main fractions, DOPW (eluted with distilled water) and DOPA (eluted with 0.1 M NaCl solution, tubes 66–111) were collected. Evaluation of the polysaccharides’ bioactivities showed that both DOPW and DOPA had excellent bioactivities. Until now, there have been few reports about the characterization of polysaccharides from NaCl elution and their potential activities. Thus, DOPA was further purified by Sephacryl S-300 chromatography to get more homogeneous polysaccharides. It was eluted with a 0.2 mol/L NaCl solution to obtain two polysaccharide fractions named DOPA-1 (tubes 23–50) and DOPA-2 (tubes 60–97), according to their molecular size (Figure 1). The yields of DOPA-1 and DOPA-2 from the crude *D. officinale* polysaccharides DOPA were 21% and 15%, respectively.

#### 2.1.2. Molecular Weight and Chemical Composition of DOPA Fractions

HPGPC was employed to determine the molecular weights of DOPA-1 and DOPA-2 (Figure S1). As shown in Table 1, the average molecular weights of DOPA-1 and DOPA-2 were determined to be 394 kDa and 362 kDa, respectively.

The monosaccharide composition of the two fractions was determined using HPLC. The results (Table 1) indicated that these two fractions had the same monosaccharide composition, and the main sugar constituents of these two polysaccharide fractions were d-mannose and d-glucose. DOPA-1 was mainly composed of d-mannose, d-glucose in the molar ratio of 5.8:1, respectively. DOPA-2 was chiefly composed of d-mannose, d-glucose in the molar ratio of 4.5:1.

#### 2.1.3. Analysis of FT-IR Spectra of DOPA Fractions

**Figure 2:**
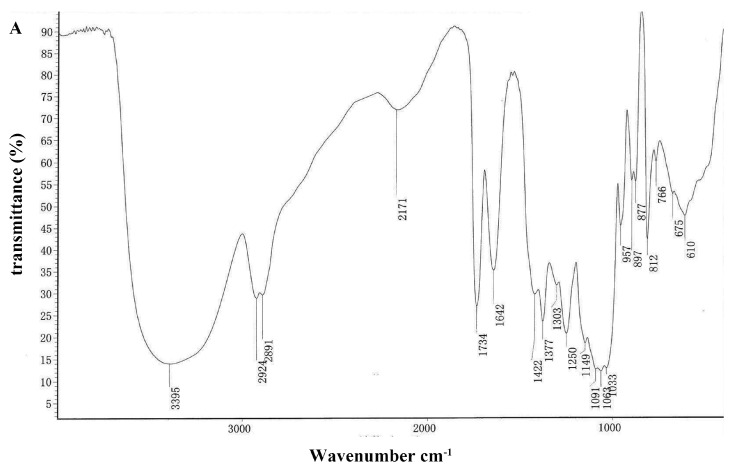

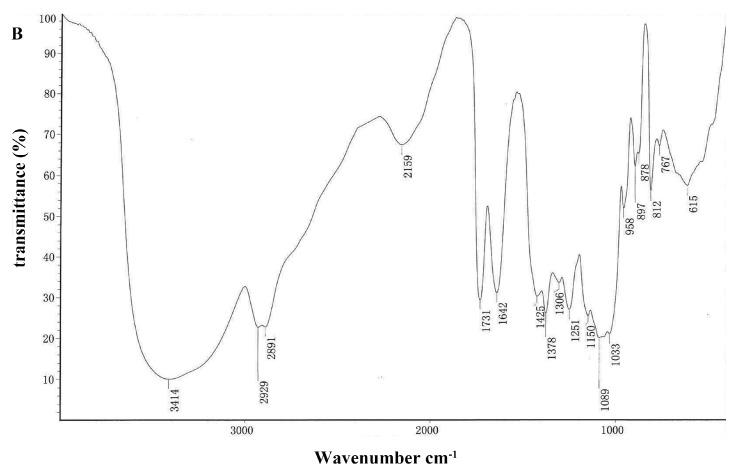
The FTIR spectra of DOPA-1 (**A**) and DOPA-2 (**B**).

FT-IR spectroscopy is typically used for the qualitative measurement of organic functional groups [23,24,25]. The FT-IR spectra of DOPA-1 and DOPA-2 are shown in Figure 2. The strong and broad absorption peaks at 3414 cm^−1^ and 3395 cm^−1^ were characteristic of *O*-H groups. The peaks at 2924, 2891, and 2929 cm^−1^ were indicative of weak C-H bond stretching vibrations. The peaks at 1734 and 1731 cm^−1^ were ascribed to valence vibration of C=O of *O*-acetyl groups. In addition, the absorption peaks at approximately 1377 and 1378 cm^−1^ could be ascribed to symmetric C-H bending vibration of the methyl groups, respectively, and the peaks at 1250 and 1251 cm^−1^ were assigned to the variable angle vibration ofthe C-O vibration of *O*-acetyl groups [26]. The peaks within the range of 1000–1200 cm^−1^ suggested the presence of C-O-C and C-O-H bonds, indicating the presence of pyranose rings [27]. In the FT-IR spectra of DOPA-1, the peak at 1064 cm^−1^ was due to the vibration of C-O at the C-4 position of a glucose residue. The peaks at 897 cm^−1^ were thought to be characteristic of β-anomeric carbon, indicating that the two fractions mainly contained β-type glycosidic linkages [28,29]. The peaks at 877 and 812 cm^−1^ were attributed to d-glucose and d-mannose in pyranose.

#### 2.1.4. Methylation and GC-MS Analysis

The glycosidic linkages of polysaccharides were determined by methylation and GC-MS analysis. According to the analysis of PMAA, the individual peaks were identified and the linkage patterns of DOPA-1 and DOPA-2 are shown in Table 2. Both of them had similar linkage patterns, mainly 1,4-linked Man*p* and 1,4-linked Glc*p*. Meanwhile, a small number of terminal groups (T-Man*p*), 1,3,4-linked Man*p*, 1,2,4-linked Man*p*, 1,4,6-linked Man*p* and 1,4,6-linked Glc*p* residues were found in DOPA-1 and DOPA-2. However, the results indicated DOPA-2 had a small amount of 1,6-linked Man*p* and 1,3,4-linked Glc*p*, which were not detected in DOPA-1. It suggested that DOPA-1 and DOPA-2 were linear glucomannans.

#### 2.1.5. Analysis of the NMR Spectra of DOPA Fractions

**Figure 3:**
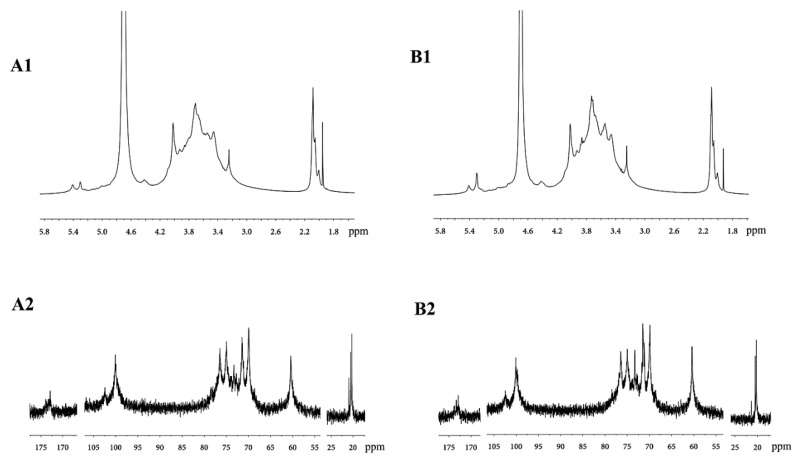
^1^H- and ^13^C-NMR spectra of DOPA-1(**A1**, **A2**) and DOPA-2 (**B1**, **B2**).

The spectra of the two purified polysaccharide fractions showed very similar signals with slight variations in peak intensity, and the signals in the spectra were weak and not well separated (Figure 3), which may be caused by the relatively high viscosities of the D_2_O solutions of DOPA-1 and DOPA-2 [26]. When comparing these chemical shifts with previously reported NMR data on similar monosaccharide compositions [10,26,30,31,32,33,34,35,36], the highest field signal at δ 2.05–2.20 belonged to the methyl group of *O-*acetyl groups in the ^1^H-NMR spectra of DOPA-1 and DOPA-2. The methyl group and ketone of the *O-*acetyl groups were detected at signals δ 20.3–21.0 and δ 172.9–173.9 in the ^13^C-NMR, respectively. The signals for the anomeric carbon at δ 100.1, and δ 102.4 were attributed to the C-1 atoms of 1,4-linked β-d-Man*p* and 1,4-linked β-d-Glc*p*. The signals from δ 60.43 to 80.26 were attributed to C-2–C-6 of the residues.

The combination of the methylation analysis and NMR spectra demonstrated that both DOPA-1 and DOPA-2 were glucomannans with *O-*acetyl groups and had a backbone consisting of 1,4-linked β-d-Man*p* and 1,4-linked β-d-Glc*p*.

### 2.2. Activation of RAW 264.7 Macrophages by D. officinale Polysaccharides in Vitro

#### 2.2.1. Effect of *D. officinale* Polysaccharides on Macrophages Viability

The cells treated with medium only had a circular morphology and a few extending pseudopodia. When RAW 264.7 cells were cultured with LPS, morphological changes were observed. Most of the cells had polygonal shapes, and the cells were larger in size than normal cells. The cells treated with *D. officinale* polysaccharides shared similarities with the cells treated with LPS in cellular morphology. The results indicated that the RAW 264.7 cells were stimulated after incubation with the polysaccharides from *D. officinale*.

**Figure 4:**
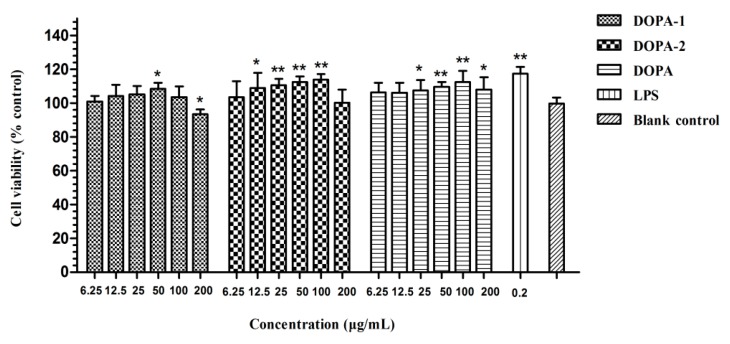
Effects of *D. officinale* polysaccharides on RAW 264.7 cell viability. The results were shown as means ± SD (*n* = 5). * *p* < 0.05, ** *p* < 0.01 compared with the blank control.

The stimulatory effect of *D. officinale* polysaccharides on RAW 264.7 cells was measured by MTT assay and it is shown in Figure 4. Compared with the blank control, LPS, DOPA-1 (50 μg/mL), DOPA-2 (12.5–100 μg/mL) and DOPA (25–100 μg/mL) exerted a significant stimulatory effect on macrophages (*p* < 0.05). MTT is an indicator of cell metabolic activity, and are suitable for analyzing proliferation and viability, and activated macrophages produce more formazan product than non-activated macrophages [37], which suggested the *D. officinale* polysaccharides might promote the viability of macrophages rather than proliferation. Although DOPA (12.5–100 μg/mL) and DOPA-2 (25–100 μg/mL) promoted RAW 264.7 cells viability with statistical significance, the polysaccharides just slightly enhanced cell viability in essence. Furthermore, the cell viability decreased at high concentrations (200 μg/mL). Similarly, DOPA-1 mildly promoted cell viability at a dose of 50 μg/mL, but inhibited cell viability at high concentrations (200 μg/mL). The other concentrations (6.25, 12.5, 25 and 100 μg/mL) did not have a stimulatory effect. Thus, the concentrations 6.25–50 μg/mL were used in the following assay in the macrophage model.

#### 2.2.2. Effects of *D. officinale* Polysaccharides on NO Production in Macrophages

The Griess test was utilized to evaluate the effects of the polysaccharides on the NO production in RAW 264.7 macrophages. As shown in Table 3, compared with the blank control, LPS significantly (*p* < 0.05) promoted NO production in macrophages. Furthermore, the polysaccharides of *D. officinale* increased the NO production in RAW 264.7 cells in a dose- and time-dependent manner, which was significantly (*p* < 0.05) different from the blank control group. In addition, NO production was detectable at a concentration of 50 μg/mL after 12 h of stimulation, which suggested that *D. officinale* polysaccharides could quickly activate macrophages. Compared with the blank control and the positive control, *D. officinale* polysaccharides still stimulated NO production at 36 h and 48 h, suggesting that the polysaccharides could maintain immunostimulatory activity for an extended period.

### 2.3. Effects of D. officinale Polysaccharides on Activivation of Splenocytes

**Figure 5:**
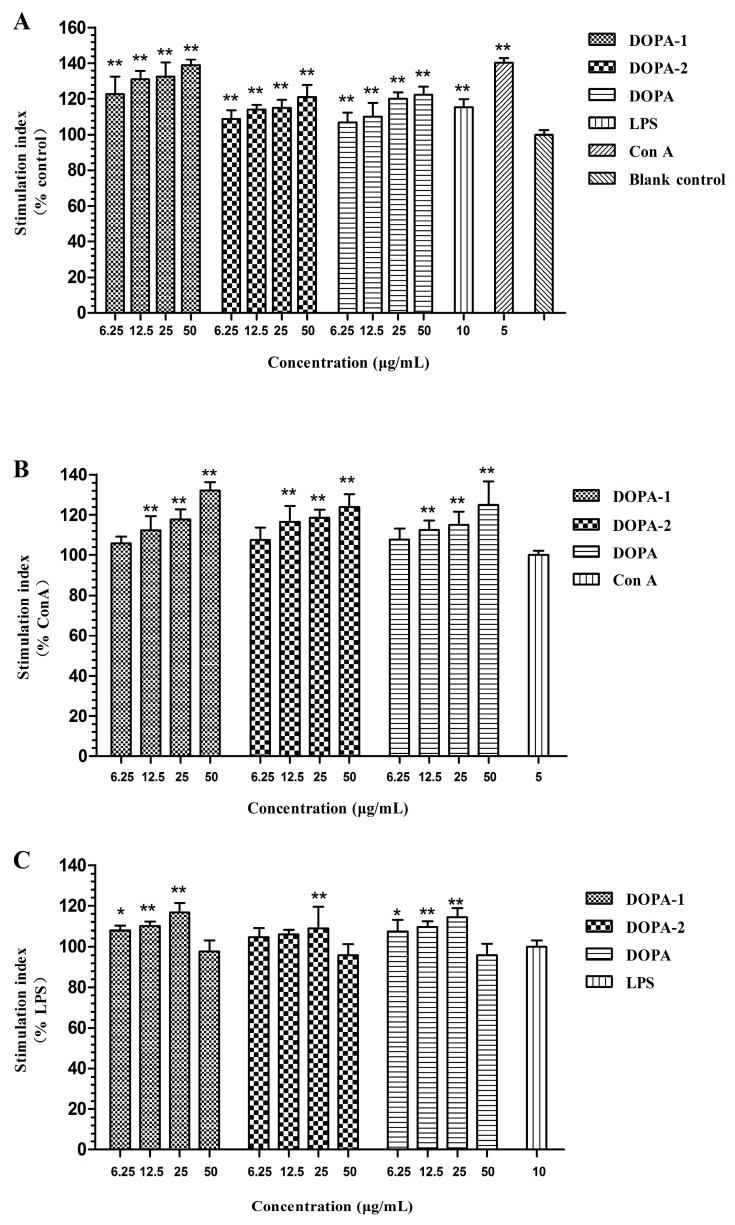
Stimulatory effects of DOPA-1, DOPA-2 and DOPA on the splenocytes. (**A**) Stimulatory effects of *D. officinale* polysaccharides on the splenocytes; (**B**) Stimulatory effects of *D. officinale* polysaccharides on the ConA-induced splenocytes; (**C**) Stimulatory effects of *D. officinale* polysaccharides on the LPS-induced splenocytes. Values were shown as means ± SD (*n* = 5). * *p* < 0.05, ** *p* < 0.01 compared to the control group.

The stimulatory effect of *D. officinale* polysaccharides on splenocytes was measured by MTT assay, and the results are displayed in Figure 5. Compared with the control group, DOPA, DOPA-1 and DOPA-2 (6.25–50 μg/mL) significantly (*p* < 0.01) stimulated splenocytes without mitogens in a dose-dependent manner. Meanwhile, they also significantly (*p* < 0.05) stimulated T-lymphocytes (ConA-induced splenocytes) in a dose-dependent manner ranging from 12.5 to 50 μg/mL. In addition, the B-lymphocytes (LPS-induced splenocytes) stimulatory effect was mildly promoted by the polysaccharides, especially at the dose of 25 μg/mL, and the stimulatory effect then decreased. MTT was used to analyse the proliferation and viability of cells. Therefore, the stimulatory effect of *D. officinale* polysaccharides on splenocytes and splenocytes with mitogens might have induced proliferation and enhanced viability of cells.

On the whole, the stimulatory effects of the polysaccharides on the splenocytes without mitogens were more noticeable than those on the splenocytes treated with ConA. The *D. officinale* polysaccharides had a mildly stimulatory effect on splenocytes treated with LPS. Among all the polysaccharides, DOPA-1 had the strongest promoting effects on stimulation of splenocytes. However, DOPA-2 had a weak effect.

### 2.4. Antioxidant Activity Assay in Macrophages Treated with H_2_O_2_

#### 2.4.1. Effect of H_2_O_2_ on the Viability of Macrophages

**Figure 6:**
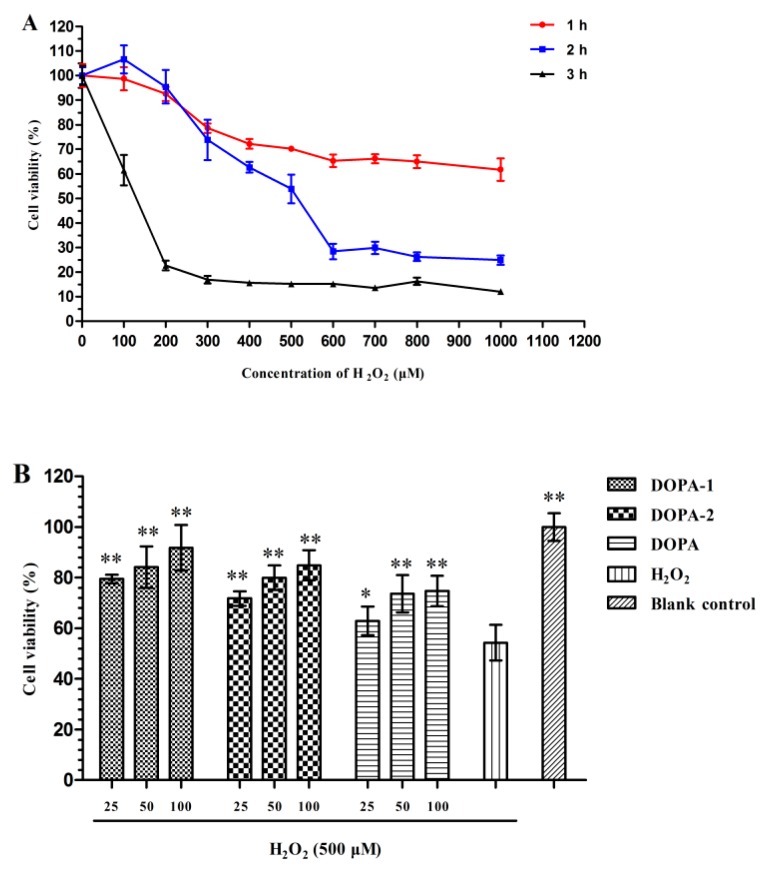
(**A**) Effects of H_2_O_2_ on the RAW 264.7 cells viability with various concentration of H_2_O_2_ for different times; (**B**) Effects of *D. officinale* polysaccharides on viability of H_2_O_2_-treated RAW 264.7 cells. Values were shown as means ± SD (*n* = 5). * *p* < 0.05, ** *p* < 0.01 compared to the H_2_O_2_-treated group.

After incubation with 100–1000 μM H_2_O_2_ over different time intervals (1, 2, or 3 h), the macrophages viability was measured by an MTT assay. The RAW 264.7 cells viability decreased in a dose- and time-dependent manner. When the cells were treated with H_2_O_2_ at doses ranging from 100 to 1000 μM for 1 h, the cell viability did not decrease dramatically. However, when incubated with H_2_O_2_ for 3 h, more than 80% of the cells were dead. As shown in Figure 6, the viability of RAW 264.7 cells treated with 500 μM H_2_O_2_ for 2 h was 53.85%. According to these results, RAW 264.7 cells treated with 500 μM H_2_O_2_ for 2 h served as the control in the remaining studies.

#### 2.4.2. Effects of *D. officinale* Polysaccharides on the Viability of H_2_O_2_-Treated Macrophages

The present study was designed to investigate whether DOPA and its fractions (DOPA-1 and DOPA-2) could exert a cytoprotective effect on macrophages treated with H_2_O_2_. The effects of the polysaccharides on the viability of H_2_O_2_-treated macrophages were analysed by an MTT assay. The cell viability decreased markedly (*p* < 0.05) after exposure to H_2_O_2_. The results, shown in Figure 6, revealed that pretreatment with DOPA, DOPA-1 and DOPA-2 (25–100 μg/mL) markedly (*p* < 0.05) promoted cell viability compared with model group. These results suggested that DOPA, DOPA-1 and DOPA-2 could protect RAW 264.7 macrophages against H_2_O_2_-induced injury.

#### 2.4.3. Effects of *D. officinale* Polysaccharides on The morphology of H_2_O_2_-Treated Macrophages

**Figure 7:**
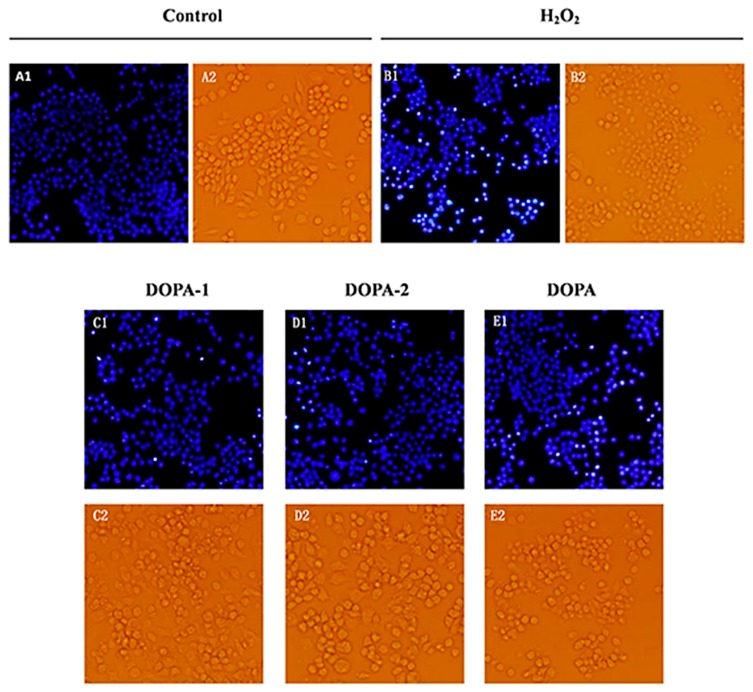
Effects of *D. officinale* polysaccharides on cell morphology of RAW 264.7 cells treated with H_2_O_2_. (1) Morphological changes of RAW 264.7 cells were observed by fluorescence microscope after staining with Hoechst 33258. (2) Morphological changes of RAW 264.7 cells were observed by phase-contrasted microscopy. (**A1**/**A2**) Cells treated with medium alone; (**B1**/**B2**) cells treated with H_2_O_2_ alone; (**C1**/**C2**) cells pretreated with DOPA-1 (100 μg/mL) prior to exposure to H_2_O_2_; (**D1**/**D2**) cells pretreated with DOPA-2 (100 μg/mL) prior to exposure to H_2_O_2_; (**E1**/**E2**) cells pretreated with DOPA (100 μg/mL) prior to exposure to H_2_O_2_.

The morphological alteration of macrophages was observed by phase-contrast microscopy. As showed in Figure 7, RAW 264.7 cells of the blank control group retained the typical macrophage-like morphology (mentioned in Section 2.2.1). RAW 264.7 cells incubated only with 500 μM H_2_O_2_ for 2 h displayed cell shrinkage, a round shape, a granuliform surface, and a large amount of cell debris. In contrast, cells pretreated with DOPA, DOPA-1 and DOPA-2 (100 μg/mL) for 24 h prior to incubation with H_2_O_2_ maintained their morphology better than the model group, showing polygonal shapes and some extending pseudopodia.

The DNA fluorescent dye Hoechst 33258 was used to further investigate the effects of *D. officinale* polysaccharides on the DNA and nuclear structure of RAW 264.7 cells treated with H_2_O_2_. As shown in Figure 7, cells treated with H_2_O_2_ alone had condensed chromatin or nuclear fragmentation and small bright, condensed dots known as apoptotic bodies, which are the biochemical indicator of apoptosis. The nuclei of the blank control were regular, with no observable condensation. In addition, pretreatment with DOPA, DOPA-1 and DOPA-2 (100 μg/mL) significantly decreased this nuclear condensation and fragmentation. The results indicated that *D. officinale* polysaccharides had a protective effect against H_2_O_2_-induced apoptosis in macrophages.

## 3. Discussion

Macrophages are the first line of defence in the host defence response after the epithelial barrier, and play an important role in innate and adaptive immune response [38,39]. Activated macrophages can kill pathogenic microorganisms, inhibit tumour growth and cancer metastasis, and clear apoptotic and mutant cells through phagocytosis and the secretion of inflammatory mediators, including cytokines, chemokines and NO [40,41]. Our results demonstrated that the polysaccharides of *D. officinale* can promote the cell viability and NO production of RAW 264.7 macrophages. Cellular and humoral immunity are an important part of immune response, characterized by T cells and B cells, respectively, which plays an important role in host defence. LPS and ConA are mitogens for B-lymphocytes and T-lymphocytes, respectively. Spleen lymphocytes induced by ConA or LPS have been used to evaluate T- or B-lymphocyte activity [42,43]. The results indicated that *D. officinale* polysaccharides could stimulate splenocytes with or without mitogen stimulation (ConA or LPS). The results demonstrated that the RAW 264.7 macrophages and splenocytes could be stimulated by the polysaccharides of *D. officinale*. On the whole, the *D. officinale* polysaccharides could promote the activity of immunocytes with statistical significance, but exerted weak effects in some indexes essentially. For example, the *officinale* polysaccharides did not promote the cell viability and NO production of macrophages, and the activity of B-lymphocytes by a large margin. The results suggested that the polysaccharides of *D. officinale* had mild immunostimulatory activity.

H_2_O_2_ is an important member of the reactive oxygen species (ROS) family [44]. It can be decomposed into a hydroxyl radical and oxygen radical to induce oxidative damage. Furthermore, H_2_O_2_ can cause prolonged damage, even after being scavenged [45,46,47]. Thus, H_2_O_2_ is a common inducer in oxidative stress cell models. Macrophages are the main targets for action of pro-oxidants. Many studies have indicated that the virulence of some bacteria triggers the death of activated macrophages by the stimulation of ROS production [48,49]. Therefore, H_2_O_2_-treated macrophages were employed in this study to examine the antioxidant activity of *D. officinale* polysaccharides. The preliminary experiments demonstrated that *D. officinale* polysaccharides significantly promoted the viability of RAW 264.7 cells induced by H_2_O_2_. In addition, the pretreatment with *D. officinale* polysaccharides significantly decreased the apoptosis induced by H_2_O_2_ and protected cell morphology and structure from H_2_O_2_-treated oxidative lesions. Based on the observed viability and morphology, our findings indicated that *D. officinale* polysaccharides could effectively attenuate H_2_O_2_-incuded cell lesions.

The bioactivity of polysaccharides is related to their molecular weight, chemical composition, glycosidic linkage, conformation, degree of branching and so on [50]. The results showed that the purified polysaccharide fractions, which had molecular weights of 394 and 362 kDa, exerted mildly immunostimulatory activity and protective effects against oxidative injury. These findings were in agreement with other reports indicating that polysaccharides with molecular weights larger than 100 kDa have excellent bioactivity. Molecular weights of the acidic polysaccharides are positively correlated to their bioactivities [51,52,53]. Notably, DOPA-1 and DOPA-2 had high mannose content. Some studies have shown that high mannose content has a positive influence on their bioactivities [54]. Both DOPA-1 and DOPA-2 contained 1,4-linked β-d-Man*p* and *O-*acetyl groups. The structural features of 1,4-linked β-d-Man*p* and *O-*acetyl groups exist in the polysaccharides of many medicinal *Dendrobium* species, such as *Dendrobium officinale*, *Dendrobium huoshanense*, *Dendrobium nobile* Lindl, and *Dendrobium tosaense* [10,26,32,36,55]. In addition, the polysaccharides showed excellent bioactivities. Therefore, the 1,4-linked β-d-Man*p* and *O-*acetyl groups are likely the main structural features contributing to the bioactivities of polysaccharides. *D. officinale* is traditionally recognized by traditional Chinese medical practitioners as the best herb among the medicinal *Dendrobium* species. Therefore, some special structural features in *D. officinale* may remain unexplored. In addition, although DOPA-1 and DOPA-2 were similar in their structural characteristics, they had different effects on biological activities. For example, DOPA-2 exerted a weak effect on splenocytes. The difference was most likely due to differences in other specific structural characteristics. Therefore, a detailed study on the structural features and mechanisms responsible for their bioactivities must be carried out to fully reveal the structure-activity relationship of *D. officinale* polysaccharides.

## 4. Materials and Methods

### 4.1. Materials and Reagents

*D. officinale* was collected from the Zhejiang Province in China. The botanical origin of plants was identified by Pro. Gang Wei. The murine macrophage cell line RAW 264.7 was obtained from the cell bank of the Chinese Academy of Science (Shanghai, China). DEAE cellulose-52 was purchased from Yuanye Biological Technology Co. (Shanghai, China), and Sephacryl S-300 was purchased from GE HealthCare Biosciences AB (Uppsala, Sweden). 3-(4,5-dimethylthiazol-2-yl)-2,5-diphenyltetrazolium bromide (MTT), lipopolysaccharide (LPS, from *Escherichia coli* serotype O111:B4), and concanavalin A (ConA, Type IV) were purchased from Sigma-Aldrich (St. Louis, MO, USA). The nitric oxide (NO) assay kit and the Hoechst staining kit were purchased from Beyotime Biotechnology (Jiangsu, China). All other reagents were of analytical grade.

### 4.2. Extraction and Isolation of Polysaccharides

#### 4.2.1. Extraction Procedures

The fresh stems of *D. officinale* were pulverized into powder in a high-speed disintegrator. To remove lipids, pigments and small molecule materials, the powder was extracted with 80% ethanol and petroleum ether successively. The residue was then extracted thrice with water. The filtrate was pooled and concentrated. Then, the concentrated solution was precipitated by adding anhydrous ethanol to a final concentration of 80% (*v*/*v*) and kept overnight at 4 °C. Next, the precipitate was dissolved in distilled water and deproteinized by the Sevag method [56]. The solution was precipitated with anhydrous ethanol and then centrifuged. After centrifugation, the precipitate was washed with anhydrous ethanol and petroleum ether in turn and then lyophilized. The crude polysaccharides were stored at 4 °C for further analyses and experiments.

#### 4.2.2. Isolation and Purification of the Polysaccharides

The crude polysaccharides were sequentially purified using DEAE cellulose-52 and Sephacryl S-300 chromatography as previously described, with slight modifications [22,53]. The crude polysaccharides were dissolved in distilled water and loaded onto an anion exchange column of DEAE cellulose-52 (pre-equilibrated with deionized water). The column was eluted with water, 0.1, 0.3, and 1.0 mol/L NaCl solutions at a flow rate of 1 mL/min. The carbohydrate content in each fraction was determined by the phenol–sulfuric acid method, and glucose was used as the standard. The fraction eluted by 0.1 mol/L NaCl solution (named DOPA) was collected, dialyzed and lyophilized. DOPA was further purified using Sephacryl S-300 chromatography and eluted with a 0.2 mol/L NaCl solution to separate two fractions (DOPA-1 and DOPA-2). The related fractions were collected, dialyzed, and lyophilized for further study.

### 4.3. Preliminary Characterization of DOPA Fractions

#### 4.3.1. Monosaccharide Composition Analysis

The monosaccharide composition of DOPA-1 and DOPA-2 was analysed by high performance liquid chromatography (HPLC). The polysaccharide samples were hydrolysed with 2 M trifluoroacetic acid solution (TFA). The hydrolysed samples and the monosaccharide standards were converted to their derivatives with 0.5 M 1-phenyl-3-methyl-5-pyrazolone (PMP). The analysis was performed on a HPLC system (Shimadzu, Kyoto, Japan). The analytical column used was an XDB-C18 column (4.6 × 150 mm, 5 μm, Agilent ZORBAX). The mobile phase was 0.05 M aqueous KH_2_PO_4_ (solvent A) and acetonitrile (solvent B).

#### 4.3.2. Molecular Weight Determination

The relative molecular weights of the two fractions (DOPA-1 and DOPA-2) were measured by high-performance gel permeation chromatography (HPGPC). The samples and the T-series dextran standards (MW: 800, 400, 200, 100, 50, 20, 10, and 5 kDa) were analysed on an Agilent 1100 series HPLC system (Palo Alto, CA, USA) equipped with the RI-101SHODEX RID detector (Tosoh, Japan) using a KS-805 column and a KS-804 column (Tosoh, Japan).

#### 4.3.4. Fourier Transform Infrared Spectroscopy Analysis

The infrared (IR) spectra of DOPA-1 and DOPA-2 were recorded by a Fourier transform infrared spectroscopy (FT-IR) spectrophotometer. The sample was ground into powder with spectroscopic grade potassium bromide (KBr) powder and then pressed into pellets for FT-IR measurement in the frequency range of 4000–500 cm^−1^ [57].

#### 4.3.5. Methylation and GC-MS Analysis

The polysaccharide fractions DOPA-1 and DOPA-2 were methylated using methyl iodide and solid sodium hydroxide in dimethyl sulfoxide according to the method reported previously [58,59]. Then, the permethylated polysaccharide samples were hydrolyzed with TFA, reduced by NaBH_4_, and then *O-*acetylated with pyridine-acetic anhydride as partially methylated alditol acetates (PMAA), which were further analyzed by GC-MS for linkage analysis. The GC-MS analysis was performed on an Agilent 7890A-5975C system (Agilent Technology, Santa Clara, CA, USA) with a HP-5 capillary column.

#### 4.3.6. Nuclear Magnetic Resonance Spectroscopy

The ^1^H-Nuclear magnetic resonance (NMR) and ^13^C-NMR spectra were recorded using a Brucker DRX-500 NMR spectrometer. DOPA-1 and DOPA-2 were dissolved in D_2_O and examined at 500 MHz at 30 °C.

### 4.4. Activation of RAW 264.7 Macrophages in Vitro

#### 4.4.1. Cell Culture

The murine macrophage cell line RAW 264.7 was maintained in RPMI-1640 medium (Gibco, Grand Island, NY, USA) containing 10% foetal bovine serum (FBS) (HyClone, Logan City, UT, USA), 100 μg/mL streptomycin and 100 U/mL penicillin (Solarbio Technology Co., Beijing, China) at 37 °C under a humidified atmosphere of 5% CO_2_.

#### 4.4.2. Cell Stimulation Assay

The stimulation of *D. officinale* polysaccharides on RAW 264.7 cells was measured using an MTT assay according to a reported method with slight modifications [37]. Briefly, the RAW 264.7 cell suspension was plated in 96-well microplates (1 × 10^5^ cells/mL), incubated 12 h and then treated with serial concentrations of polysaccharides DOPA, DOPA-1, and DOPA-2 (6.25, 12.5, 25, 50, 100, 200 μg/mL) for 24 h. Cells treated with equal volumes of RPMI-1640 medium and LPS solution (0.2 μg/mL) were used as a vehicle control and a positive control. After treatment, cells were incubated with the MTT solution (5 mg/mL) for another 4 h, and then the medium was discarded. The formazan crystals were dissolved using 100 μL of dimethyl sulfoxide (DMSO). The absorbance was read at 570 nm on a microplate reader (Bio-Rad, Hercules, CA, USA). The stimulation index was expressed as the ratio of the absorbance values of the treatment group to values of the vehicle control group.

Stimulation index = OD _experimental_/OD _control_ × 100% (1)

#### 4.4.3. Assay of the Nitric Oxide (NO) Production of Macrophages

The Griess reaction was applied to evaluate the NO production of the cells [60]. RAW 264.7 cells were treated with different concentrations of DOPA, the DOPA fractions, LPS and RPMI-1640 medium, similarly to the above treatments. After incubation, the supernatant was collected and reacted with an equal volume of Griess reagent at room temperature for 15 min. The absorbance was read at 570 nm, and the nitrite (NaNO_2_) was used as a standard.

### 4.5. Activation of Splenocytes in Vitro

The stimulation of *D. officinale* polysaccharides on splenocytes was measured using an MTT assay as described previously [37,61,62]. Spleens collected from male BALB/c mice were minced using surgical scissors. The spleen fragments were grinded through a stainless steel cell strainer into RPMI-1640 medium (without 10% FBS). The cell suspension was centrifuged at 110× *g* for 4 min, and then the supernatant was removed. The recovered spleen cells were resuspended in lysis buffer (0.15 M NH_4_Cl, 0.01 M KHCO_3_, 0.0001 M EDTA-2Na) to remove the erythrocytes. The cells were washed twice with phosphate-buffered saline (PBS) and resuspended in RPMI-1640 medium. The viability of the splenocytes was over 95%, according to the trypan blue dye exclusion test. Cells (3 × 10^6^ cells/mL) were plated in 96-well plates and then treated with 6.25, 12.5, 25, or 50 μg/mL polysaccharides, respectively, or with a polysaccharide (6.25, 12.5, 25, 50 μg/mL) solution containing LPS (10 μg/mL) or ConA (5 μg/mL). Cells treated with RPMI-1640 medium were used as a vehicle control. After incubating for 72 h, the MTT solution was added to each well and then further incubated for another 4 h. Subsequently, the cell suspension was centrifuged at 309× *g* for 15 min, and the medium was removed. One hundred microliters of DMSO was added to dissolve the formazan crystals. The absorbance was read at 570 nm. The stimulation index was calculated by the following equation:

Stimulation index = OD _experimental_/OD _control_ × 100% (2)

### 4.6. Antioxidant Activity Assay in Macrophages Treated with H_2_O_2_

#### 4.6.1. Assessment of Cell Viability

The cell viability was determined using an MTT assay [44]. Briefly, RAW 264.7 cells were cultured at a density of 1 × 10^5^ cells/mL in 96-well plates overnight and were treated with indicated concentrations of DOPA, DOPA1 and DOPA-2 (25, 50, 100 μg/mL) for 24 h. The cells of blank control group and model group were incubated with an equal volume of RPMI-1640 medium. After treatment, the medium was discarded. Then, all of the cells were treated with H_2_O_2_ (500 μmol/L) for 2 h, except the blank control cells were treated with an equal volume of RPMI-1640 medium without H_2_O_2_. The MTT solution was added to each well, and the plates were incubated for 4 h at 37 °C. The medium was discarded, and 100 μL of dimethyl sulfoxide (DMSO) was added to the wells to solubilize the crystals. Finally, the absorbance was measured by a microplate reader at 570 nm. The cell viability was calculated by the following equation:

Cell viability (%) = OD _experimental_/OD _blank control_ × 100% (3)

#### 4.6.2. Morphological Observation

The morphological changes of RAW 264.7 cells were observed under a phase-contrast microscope (BX51, Olympus Optical Co. Ltd., Tokyo, Japan). RAW 264.7 cells were seeded in 12-well plates at a density of 1 × 10^5^ cells/mL. After the pretreatment, the cells were stained with Hoechst 33258 dye staining for 30 min at room temperature in the dark and then observed under a fluorescence microscope (BX51, Olympus Optical Co. Ltd.).

### 4.7. Statistical Analysis

All experiments were repeated at least three times. All values are expressed as the mean ± standard deviation (SD). Statistical significance was determined by one-way analysis of variance (ANOVA). *p* < 0.05 was considered to be statistically significant. All statistical analyses were conducted using SPSS for Windows, Version 19.0 (SPSS, Chicago, IL, USA).

## 5. Conclusions

In this study, crude polysaccharides were successfully extracted from the stem of *D. officinale*. DOPA and two fractions (DOPA-1, DOPA-2) were isolated and purified. Their structural characteristics and bioactivities were investigated. These two polysaccharide fractions mainly comprised d-mannose and d-glucose. Their molecular weights were 394 kDa and 362 kDa, respectively. The combination of the methylation analysis and spectra analysis (FT-IR, ^1^H- and ^13^C-NMR) demonstrated that both DOPA-1 and DOPA-2 were glucomannans with *O-*acetyl groups and had a backbone consisting of 1,4-linked β-d-Man*p* and 1,4-linked β-d-Glc*p*. Furthermore, the bioactivity studies demonstrated that *D. officinale* polysaccharides could slightly promote the cell viability and NO production of RAW 264.7 macrophages, and exert stimulatory effects on splenocytes, T-lymphocytes and B-lymphocytes. In addition, *D. officinale* polysaccharides exerted significant protective effects against H_2_O_2_-induced oxidative injury in RAW 264.7 macrophages. Thus, the *D. officinale* polysaccharides possessed mild immunostimulatory activity and antioxidant activity.

## Figures and Tables

**Table 1:** Contents of carbohydrate and monosaccharide compositions for polysaccharide fractions from *D. officinale*.

Sample	Carbohydrate (%)	Molecular Weight (kDa)	Monosaccharide Composition (Molar Ratio)	
			d-Mannose	d-Glucose
DOPA-1	93.80%	394	5.8	1
DOPA-2	91.60%	362	4.5	1

**Table 2:** Methylation analysis and of DOPA-1 and DOPA-2.

Retention Time (min)	Linkage Pattern	Major Mass Fragments (*m*/*z*)	Peak Area Percentage (%)	
			DOPA-1	DOPA-2
10.69	T-Man*p*	102, 117, 129, 145, 161, 205	4.08	2.43
12.44	1,4-linked Man*p*	101, 113, 117, 129, 131, 143, 161, 173, 233	79.63	78.49
12.53	1,4-linked Glc*p*	101, 113, 117, 129, 131, 143, 161, 173, 233	14.39	16.99
12.73	1,6-linked Man*p*	101, 117, 129, 161, 189, 233	-	0.22
13.35	1,3,4-linked Man*p*	118, 129, 160, 143, 185, 203, 231, 305	0.48	0.35
13.44	1,3,4-linked Glc*p*	118, 129, 160, 143, 185, 203, 231, 305	-	0.15
13.71	1,2,4-linked Man*p*	113, 130, 143, 172, 190, 231	0.51	0.57
14.05	1,4,6-linked Man*p*	101, 117, 127, 142, 159, 201, 261	0.47	0.37
14.14	1,4,6-linked Glc*p*	101, 117, 127, 142, 159, 201, 261	0.44	0.43

**Table 3:** Effects of *D. officinale* polysaccharides on the production of NO in RAW 264.7 cells.

**A**	**Concentration (μg/mL)**	**NO Production (μM)**				
		**Blank Control**	**LPS**	**DOPA-1**	**DOPA-2**	**DOPA**
	0	2.38 ± 0.53				
	0.2		20.28 ± 0.38 **			
	6.25			3.59 ± 0.33 *	4.12 ± 0.57 **	3.42 ± 0.36 *
	12.5			4.58 ± 0.74 **	4.86 ± 0.42 **	3.60 ± 0.73 **
	25			5.11 ± 0.52 **	5.17 ± 0.54 **	3.89 ± 0.77 **
	50			6.89 ± 0.48 **	7.43 ± 0.52 **	5.24 ± 0.78 **
**B**	**Time (h)**	**NO Production (μM)**				
		**Blank Control**	**LPS**	**DOPA-1**	**DOPA-2**	**DOPA**
	12	1.77 ± 0.04	3.59 ± 0.15 **	2.75 ± 0.15 **	2.98 ± 0.09 **	1.75 ± 0.05
	24	1.70 ± 0.58	13.11 ± 1.53 **	3.29 ± 0.46 *	3.45 ± 0.34 **	2.25 ± 0.47
	36	1.94 ± 0.42	19.53 ± 0.43 **	4.23 ± 0.65 **	4.53 ± 0.73 **	3.26 ± 0.46 **
	48	2.12 ± 0.74	19.76 ± 0.45 **	6.13 ± 0.61 **	7.08 ± 0.67 **	4.97 ± 0.31 **

(**A**) The cells were treated with *D. officinale* polysaccharides (6.25–50 μg/mL) or LPS (0.2 μg/mL) for 48 h; (**B**) RAW 264.7 cells were incubated with *D. officinale* polysaccharides (50 μg/mL) or LPS (0.2 μg/mL) for 12, 24, 36 and 48 h. The results were shown as means ± SD (*n* = 4). * *p*< 0.05, ** *p* < 0.01 compared with the blank control.

## Supplementary Materials

Supplementary materials can be accessed at: http://www.mdpi.com/1420-3049/21/6/701/s1.
