# Supplementary material for: Purification, Characterization and Biological Activity of Polysaccharides from Dendrobium officinale
Source: Molecules. 2016 May 30;21(6):701. doi: 10.3390/molecules21060701 (PMC6272863; doi:10.3390/molecules21060701)
Supplement: Supplementary file 1 [file molecules-21-00701-s001.pdf]

Supplementary Materials: Purification, Characterization and Biological Activity of Polysaccharides from *Dendrobium officinale*

Kaiwei Huang, Yunrong Li, Shengchang Tao, Gang Wei, Yuechun Huang, Dongfeng Chen and Chengfeng Wu

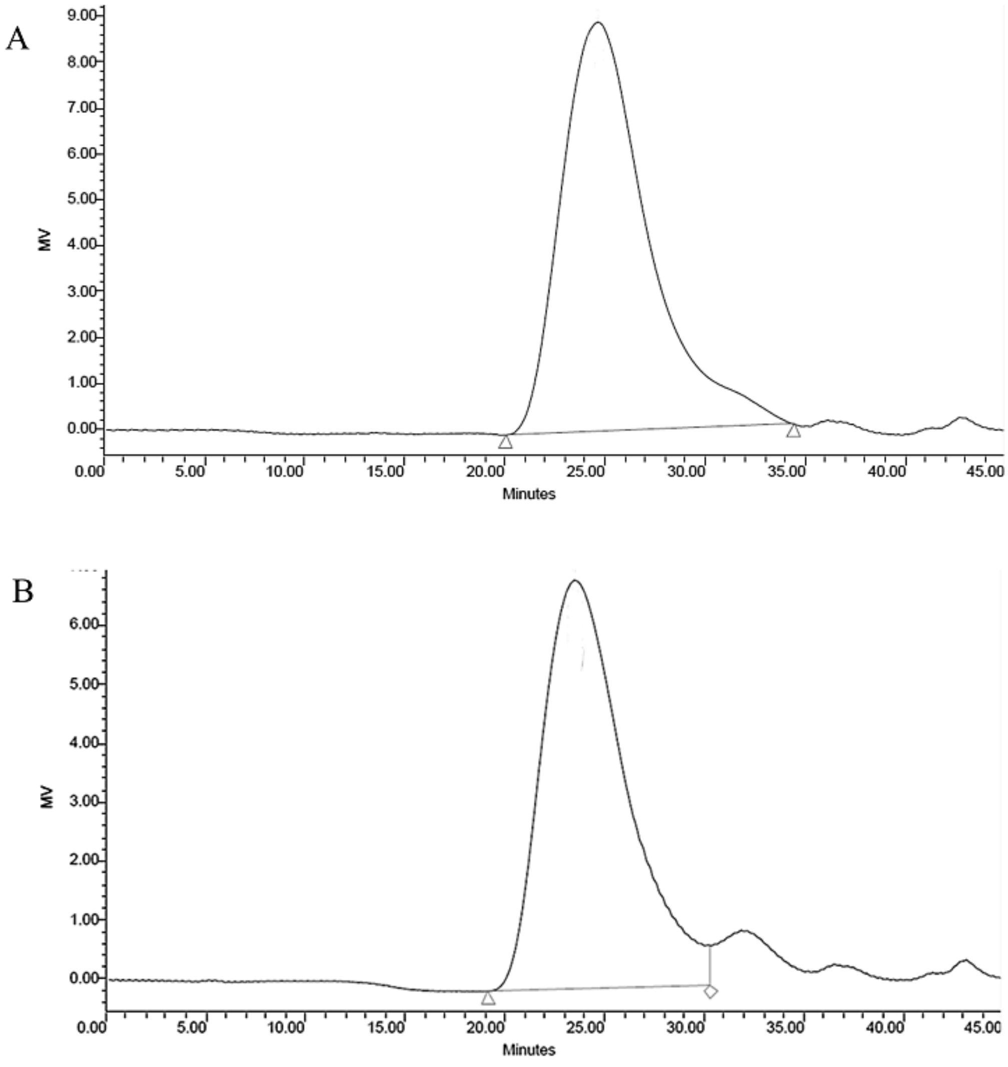

Figure S1. The HPGPC spectra of DOPA-1 (**A**) and DOPA-2 (**B**).

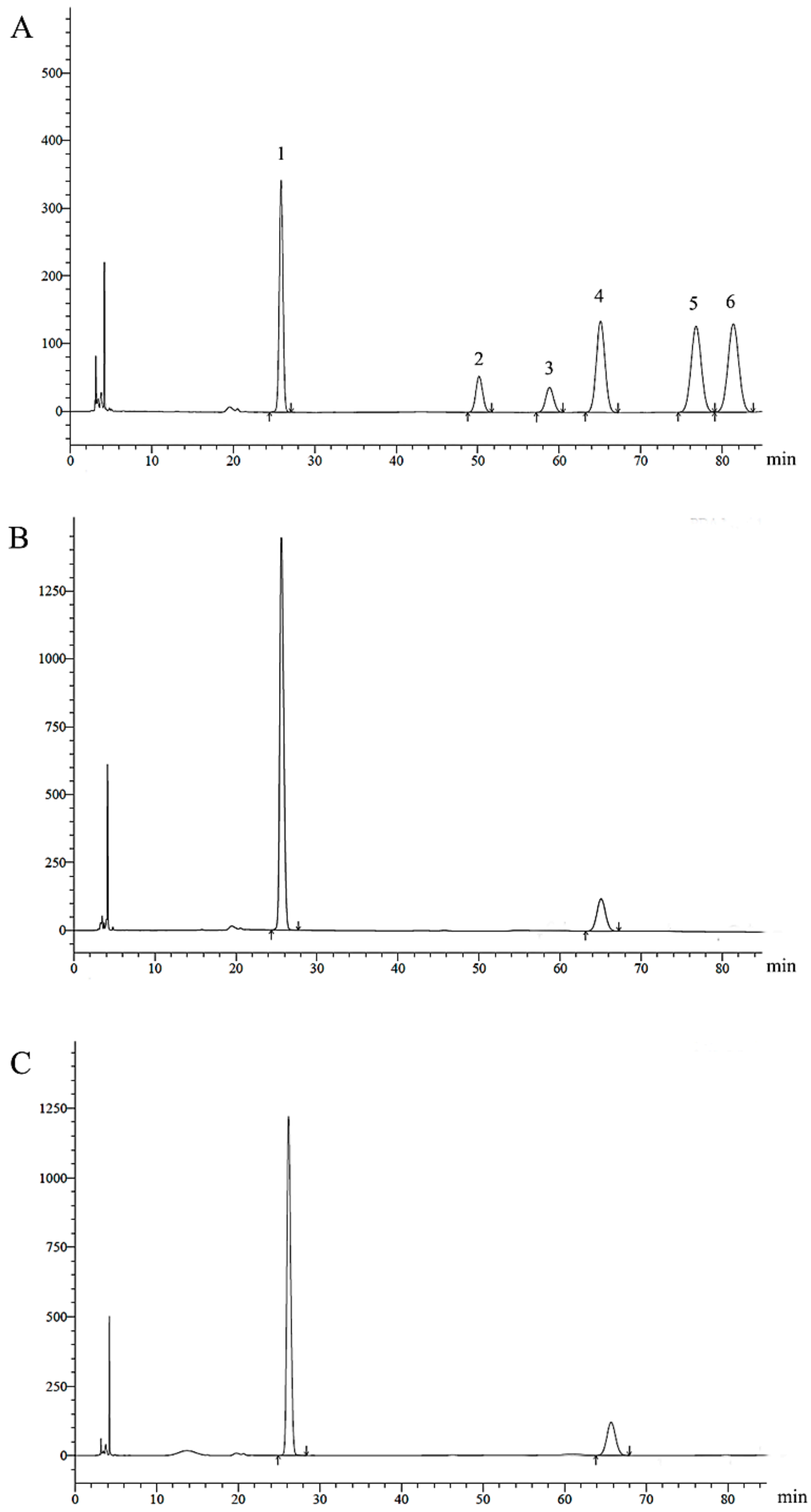

Figure S2. The HPLC chromatograms of PMP derivatives of six standard monosaccharides (Peaks: 1. Mannose, 2. Glucuronic acid, 3. Galacturonic acid, 4. Glucose, 5. Galactose, 6. Xylose) (**A**), monosaccharide component in DOPA-1 (**B**) and monosaccharide component in DOPA-2 (**C**).
